# Archery's signature: an electromyographic analysis of the upper limb

**DOI:** 10.1017/ehs.2022.20

**Published:** 2022-05-26

**Authors:** Tabitha Dorshorst, Gillian Weir, Joseph Hamill, Brigitte Holt

**Affiliations:** 1 Department of Anthropology, University of Massachusetts, Amherst, MA, USA; 2 Biomechanics Laboratory, University of Massachusetts, Amherst, MA, USA

**Keywords:** Archery, EMG, humeral asymmetry

## Abstract

**Non-technical summary:**

Bow and arrow technology plays a significant role in the recent evolutionary history of modern humans, but limitations of preservation make it challenging to identify archaeological evidence of early archery. Since bone structure can change in response to muscle force, archers of the past can potentially be identified through analysis of upper arm bones. However, there is limited research on how archery impacts upper limb musculature. This study offers initial insights into how archery impacts humeral musculature and highlights the need for additional research focused on archery's direct impact on humeral morphology.

**Technical summary:**

Humeral morphology has been used to support behaviour reconstructions of archery in past populations. However, the lack of experimental research concerning the impacts that archery has on the upper limb weakens skeletal morphological approaches. The goal of this study was to determine how archery impacts the activation of upper limb musculature. More specifically, this study tested: (a) whether the relative muscle activations are similar between arms; and (b) what muscles were activated on the dominant (draw) arm compared with the non-dominant (bow) arm. Data on upper arm muscle activation were collected bilaterally for nine archers using surface electromyography (EMG). Results show similar levels of muscle activation bilaterally with different muscles being activated in each arm. There were significantly higher integrated EMG and peak muscle activations of the biceps brachii muscles in the draw arm compared with the bow arm. In contrast, the lateral deltoid and the triceps brachii muscles had significantly higher integrated EMG and peak muscle activations on the bow arm compared with the draw arm. This work offers initial insights into how archery impacts humeral musculature and highlights the need for additional research focused on archery's direct impact on humeral morphology.

## Graphical abstract

**Figure:**
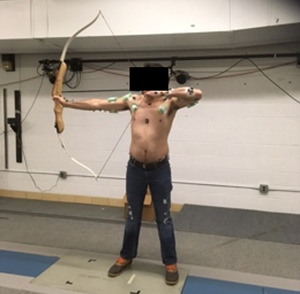

**Social media summary:** Archery's impact on humeral musculature and the need for research on archery's direct impact on humeral morphology.

## Introduction

Bow and arrow technology plays a significant role in the recent evolutionary history of modern humans and is universally present across human societies, arguably as early as 61.7 ± 1.5 thousand years BP (Lombard, 2005, 2011; Lombard & Haidle, 2012). Lombard and Shea (2021) suggest that the bow and arrow may represent the earliest evidence for complex projectile weapons, and their versatility allowed humans to be successful hunters in diverse ecological contexts, such as arctic, woodland and desert biomes (Shea, 2006; Williams et al., 2014). Archery is associated with more advanced cognition than earlier projectile tools (e.g. spear throwing), in part because it requires the manufacture of two tools – the bow and the arrows – that rely on each other to function (Ambrose, 2010; Sisk and Shea, 2011). Arrow-shooting tasks have also been shown to require more enhanced executive functions in the brain compared with spear-throwing tasks (Williams et al., 2014), further supporting the claim that archery is associated with cognitive complexity. Therefore, the appearance of archery in the archaeological record demarcates an important shift in the cognitive complexity that defines modern humans (Osiurak & Massen, 2014; Williams et al., 2014).

Limitations of preservation make identifying archaeological evidence of early archery challenging. Early bows and arrows were typically made from organic materials that do not preserve well over time (Whitman, 2017). Durable stone arrowheads are the most demonstrative archaeological evidence of archery in the deep past because stone points preserve better than the organic material of the bow and arrow shafts. Arrowheads appear similar in structure to other types of projectile points, such as spearheads and dart points, which makes it difficult to differentiate among them (Shea, 2006). The oldest proposed arrowheads in the archaeological record were excavated from Sibudu Cave in Kwazulu–Natal, South Africa, dating to 61.7 ± 1.5 thousand years BP (Lombard, 2011; Lombard & Haidle, 2012). The next evidence of archery does not appear until c. 12,000 years BP in the Levant region (Johannes, 2004). Unfortunately, both sites offer only indirect evidence of archery in the form of possible arrowheads. On their own, projectile points offer weak, indirect evidence of archery since tools were often used for multiple purposes. Without a bow, arrowheads can only imply archery. The first archaeological evidence of a bow does not appear until the famous Holmegaard Bows in Denmark, c. 8,000 years BP (Knecht, 1997; Whitman, 2017). The limited and indirect nature of archaeological material evidence for early archery underscores the importance of finding other sources of data to support hypotheses about archery in past populations. Analysis of skeletal morphology is often used to reconstruct past behaviours (Larsen, 2015; Ruff et al., 2018); in particular, examining signatures of archery on the upper limb bones could provide additional information and insights as to the origins of archery.

The impact of archery on humeral cross-sectional asymmetry (measured from the total cortical area) has been inferred to support behaviour reconstructions from past populations (Peterson, 1998; Rhodes & Knüsel, 2005). Given that bone remodels in response to mechanical loads (Wolfe's Law or bone functional adaptation; Taylor et al., 2009; Larsen, 2015; Ruff et al., 2018), it is reasonable to assume that the repeated use of a bow and arrow would impact upper limb skeletal morphology in predictable ways. Even though there is minimal experimental evidence articulating how archery impacts the upper limb bones, it has been suggested that archery lowers asymmetry in humeral robusticity (Schmitt et al., 2003; Rhodes & Knüsel, 2005).

Archery is a bimanual activity that requires the dominant arm (often referred to as the draw arm) to draw back the bowstring, while the non-dominant arm (also called the bow arm) holds the bow. Changes in hunting strategies towards the end of the Early Upper Paleolithic involved shifts from unimanual weapons such as throwing spears to bimanual weapons such as bows (Schmitt et al., 2003). Early modern human males are characterised as having high levels of humeral cross-sectional asymmetry (Trinkaus et al., 1994; Trinkaus & Churchill, 2002); however, subsequent decreases in humeral asymmetry are observed during the Mesolithic (10,500–6050 years BP; Sládek et al., 2016). Thus, the decrease in humeral cross-sectional asymmetry in males from the Upper Paleolithic to the Mesolithic could be a result of increased loading on the non-dominant arm from the use of bows. Similarly, the introduction of the bow during the Mississippian Period in North America could explain the decrease in male humeral cross-sectional asymmetry observed during the Mississippian period (Bridges, 1989). In a more recent context, the humeri of archers recovered from the *Mary Rose* ship that sank in AD 1545 bear no evidence of significant differences in humeral size asymmetry, suggesting equal use of both arms (Stirland, 1992).

Humeral diaphyseal shape, measured as the ratio of antero-posterior to medio-lateral bending strength (*I_x_*/*I_y_* ratio), may be used to infer patterns of activity (Trinkaus et al., 1991), including archery (Rhodes & Knüsel, 2005). Bone shape is impacted by strains placed on the bone due to mechanical loading, which includes external impact forces (e.g. ground reaction forces) and internal muscle forces (Schipilow et al., 2013). Skeletal muscles place force on the bones either directly or indirectly through the activation of adjacent muscles, with the largest force occurring during locomotion and lifting activities (Avin et al., 2015; Burr, 1997). Changes in the force production of muscles are suggested to coincide with changes in bone properties, such as strength and shape (Burr, 1997). As a bimanual activity, archery requires each arm to perform unique movements that activate different muscles. Generally, an individual's dominant arm pulls back on the bowstring, hence being called the ‘draw arm’, while the non-dominant arm, or ‘bow arm’, holds the bow in position (Peterson, 1998). Rhodes and Knüsel (2005) hypothesise that increased anterior–posterior (A/P) bending in the humerus that results in A/P-oriented humeral shape is directed largely by muscles responsible for flexion and extension (i.e. triceps brachii, biceps brachii and brachialis muscles), while medial–lateral (M/L) bending in the humerus that results in increased M/L humeral shape is directed largely by the deltoid muscle. As an example of this, Rhodes and Knüsel (2005) note that individuals from the Towton, England (fifteenth century) sample exhibit increased average medio-lateral humeral shape in the left arm. They posit that this difference in diaphyseal shape could be a result of using a weapon requiring both arms, such as a bow, and more particularly a longbow – the most common bow used in England at this time (Gunn, 2010). The muscles involved in holding a bow (e.g. deltoid muscle) could, therefore, contribute to M/L bending in the humerus. There is, however, limited research examining which muscles are activated bilaterally during archery.

Given the well-documented ability of bones to adapt their shape and size in response to mechanical loading (Larsen, 2015; Ruff et al., 2018), it is reasonable to assume that the repeated action of practising archery would impact humeral morphology. Unfortunately, the impacts of archery on the upper limb lack experimental research. In this study, we used surface electromyographic (sEMG) analyses to investigate the impacts of archery on the activation of upper limb musculature. We tested: (a) whether the relative muscle activations are similar between arms; and (b) what muscles were activated on the dominant (draw) arm compared with the non-dominant (bow) arm. Through the study of functional asymmetry in muscle activation during archery, this study aims to better contextualise the loading environment of the upper limbs relative to functional interpretations of the skeletal record.

## Methods

### Participants

Participants were recruited throughout Amherst, Massachusetts, via flyers posted at the University of Massachusetts Amherst campus and local gymnasiums. Nine males (mean ± SD: age, 22 ± 4 years; height, 1.79 ± 0.07 m; weight, 78.6 ± 8.9 kg) who reported no major upper limb injuries or surgeries within the past year participated in a single motion capture and electromyography testing session in the University of Massachusetts Biomechanics Laboratory. Participants self-reported their experience level with archery. Beginners were categorised as having less than one year of archery experience, while participants with more than five years of archery were considered experienced. Table 1 shows all participant demographics.

**Table 1.** Participant demographics and self-reported years of experience

Subject ID	Age (years)	Height (m)	Mass (kg)	Draw (dominant) arm	Years of experience
P01	28	1.75 m	78.0 kg	Left	<1 year: beginner
P02	20	1.74 m	77.1 kg	Right	8 years: experienced
P03	23	1.80 m	81.6 kg	Right	10 years: experienced
P04	23	1.87 m	81.6 kg	Right	15 years: experienced
P05	21	1.80 m	68.0 kg	Right	9 years: experienced
P06	20	1.70 m	63.5 kg	Right	9 years: experienced
P07	20	1.73 m	79.3 kg	Right	<1 year: beginner
P08	18	1.80 m	94.3 kg	Right	<1 year: beginner
P09	31	1.91 m	83.9 kg	Right	<1 year: beginner

The study's protocol was approved by the University of Massachusetts Institutional Review Board and all participant completed an informed written consent prior to participation.

### Experimental setup

Surface electromyography data were collected bilaterally for eight muscles (latissimus dorsi, pectoralis major, biceps brachii, deltoid anterior fibres, deltoid lateral fibres, deltoid posterior fibres, triceps brachii long head and triceps brachii lateral head) on the upper limbs and torso using a 16-channel Delsys Trigno Wireless electromyography (EMG) system at a sampling rate of 2000 Hz. These specific muscles were indicated as key muscles involved in archery (Peterson, 1998; Rhodes & Knüsel, 2005) and are close enough to the surface of the skin to be measured with sEMG. Sensors were placed on the skin over the muscle bellies according to SENIAM guidelines (Hermens & Freriks, 1997; Konrad, 2006).

Kinematic data were collected in the University of Massachusetts Biomechanics Laboratory using an 11-camera motion capture system (Qualisys Inc., Gothenburg, Sweden), sampling at 200 Hz. Participants used a standard recurve bow for all trials and three retroreflective markers were placed on the recurve bow and one marker on the back of the arrow.

### Protocol

Electromyography measures muscle activation but cannot be directly compared across different muscles or individuals without first being standardised (Chen et al., 2018). Maximum voluntary contractions (MVCs) were collected in order to standardise the EMG signals. Maximum voluntary contractions act as a maximum strength test and represent the maximum effort capacity of a muscle (Chen et al., 2018). The EMG signal measured is then set as a percentage of the MVC. Following sEMG sensor placement, three MVC trials were collected for each muscle, bilaterally (Konrad, 2006). Participants were asked to use maximum effort to perform a task (outlined in Konrad, 2006) designed to isolate the targeted muscle for 10 seconds (Boettcher et al., 2008). Throughout maximal contractions, participants were verbally encouraged to use their maximum force. Participants alternated between right and left arm tasks for all trials to avoid fatigue. The largest value from the three trials for each muscle was used as each individual's maximum voluntary contraction for the targeted muscle. This value was used to standardise individual muscle activation so that all results are reported as a percentage of their MVC.

**Figure 1.**
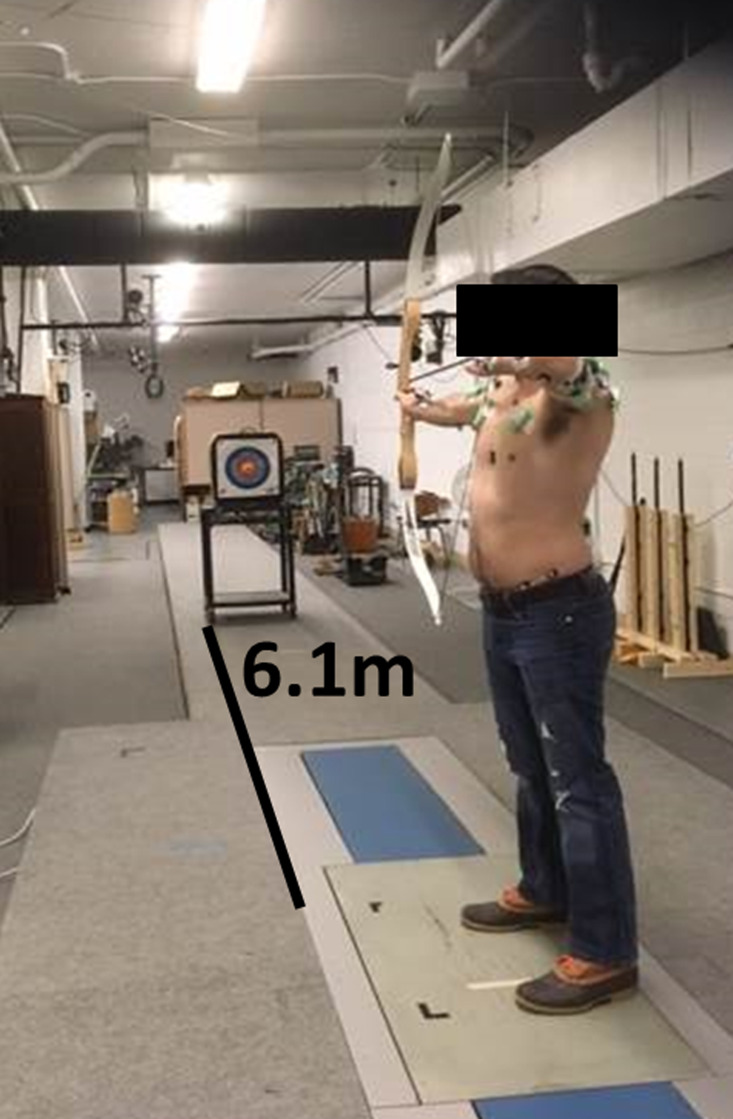
Image of a participant at full draw with the target placed at chest level 6.1 m away from the participant's front foot.

After MVC collection, participants were instructed to stand in the centre of the data collection area in their preferred stance with a target placed at chest level, 6.1 m from the participant's front foot (Figure 1). The target distance of 6.1 m was selected owing to space limitations and safety concerns. To avoid variation in muscle activation owing to bow type or draw weight, all participants used the same bow, a standard recurve bow with a draw weight of 0.45 kg. Left-hand dominant individuals were given a left-handed recurve bow made from the same material and same draw weight. The draw weight was set at 0.45 kg to ensure each archer was fully capable of drawing the bowstring back completely without compromising form or risk of injury. After each participant warmed up with their preferred methods, data were collected for 10 trials. Participants were allowed to take as much time as required to aim and release the arrow for each trial. Once the arrow was released, participants retrieved their arrow and were given the option to rest before the next trial to minimise muscle fatigue.

### Data analysis

Kinematic markers were identified in Qualisys Track Manager (Qualisys Inc., Gothenburg, Sweden) and processed using Visual 3D software (CMotion Inc., Rockville, MD). The tracked data were filtered with a low-pass Butterworth filter at 4 Hz based on a residual analysis and visual inspection. The process of shooting the bow and arrow is made up of two components, the draw phase and the full draw event. The draw phase begins when the distance between the marker placed on the arrow and the marker placed on the middle of the bow first deviates more than one standard deviation from the starting position and ends when participants reach the full-draw event. The full draw event refers to the moment participants reached maximum distance between the marker on the arrow and the middle bow marker.

Surface electromyographic data were processed through a customised MATLAB (Version 2018b) program. Any environmental noise or direct current offset recorded during data collection was removed and negative amplitudes of the signal were converted to positive amplitudes through a full-wave rectification (Konrad, 2006). Then a bandpass filter from 20 to 500 Hz removed any additional noise seen in the signal, such as heartbeat signals. Finally, the signal was filtered with a low-pass Butterworth filter at 6 Hz to create a linear envelope (Merletti & di Torino Politecnico, 2015). Individual muscle activation amplitude for each muscle was standardised to their MVC. Peak muscle activation and integrated EMG (iEMG) were analysed during the draw phase, since this was the phase with the highest amplitude of muscle activity.

### Statistical analysis

A Wilcoxon signed-rank statistical test performed in R (version 4.0.5) showed no significant differences between muscle activation of experienced and beginner archers. Therefore, to increase sample size, participants were pooled together regardless of experience. Differences in muscle activation were assessed with a non-parametric Wilcoxon signed-rank statistical test using R (version 4.0.5). Results were considered statistically significant at the *p* < 0.05 level.

## Results

**Figure 2.**
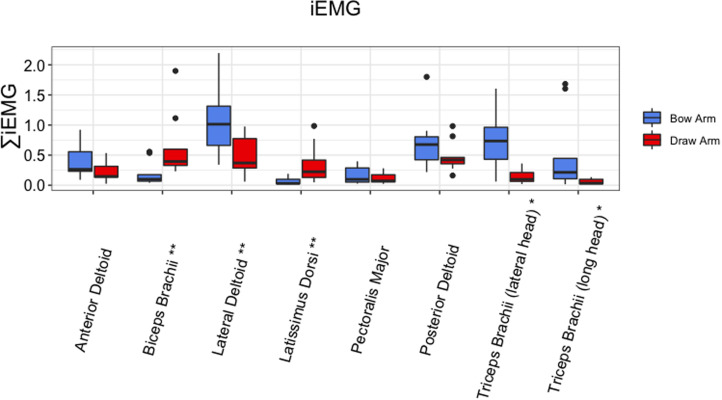
Average integrated EMG (iEMG) during the draw phase of the non-dominant (bow) arm and the dominant (draw) arm. *Statistical significance between the bow arm and the draw arm at *p* < 0.05; **statistical significance between the bow arm and the draw arm at *p* < 0.01.

**Figure 3.**
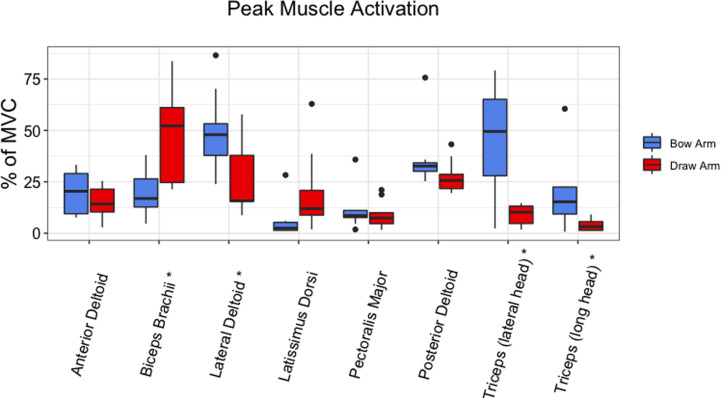
Average peak muscle activation as a percentage of isometric maximum voluntary contraction (MVC) of the non-dominant (bow) arm and the dominant (draw) arm during the draw phase. *Statistical significance between the bow arm and the draw arm at *p <* 0.05.

Statistically significant differences in the area under the curve of the rectified EMG signal, referred to as iEMG, between the draw arm and the bow arm were observed for several muscles (Figure 2). The iEMG values for the latissimus dorsi (*p* = 0.004) and biceps brachii (*p* = 0.004) were greater in the draw arm than the bow arm. In contrast, greater iEMG values for the lateral deltoid (*p* = 0.004) and triceps brachii (long head *p* = 0.019 and lateral head *p* = 0.023) were observed in the bow arm. There were no statistically significant differences between arms observed for posterior deltoid (*p* = 0.164) and anterior deltoid (*p* = 0.301). Peak sEMG amplitudes (Figure 3) mirrored those of iEMG. The peak sEMG amplitude of the biceps brachii (*p* = 0.019) were greater in the draw arm than the bow arm. Similar to the iEMG results, the peak amplitudes for the deltoid (lateral) (*p* = 0.039) and the triceps brachii (long head *p* = 0.016 and lateral head *p* = 0.016) were greater in the bow arm. Tables with values for both iEMG and peak sEMG amplitude can be found in the Supplementary Material.

**Figure 4.**
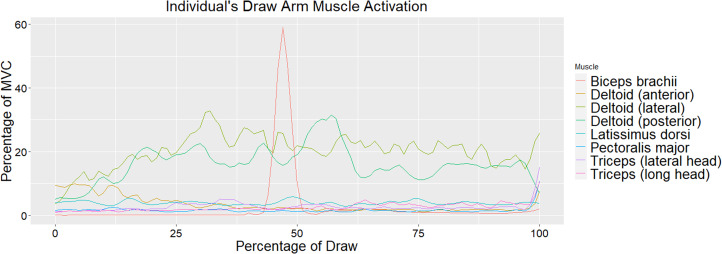
An individual's draw arm muscle activation as a percentage of MVC of the bow and draw arm as a percentage of the draw. Zero represents the start of the draw phase and 100 represents the individual at full draw.

**Figure 5.**
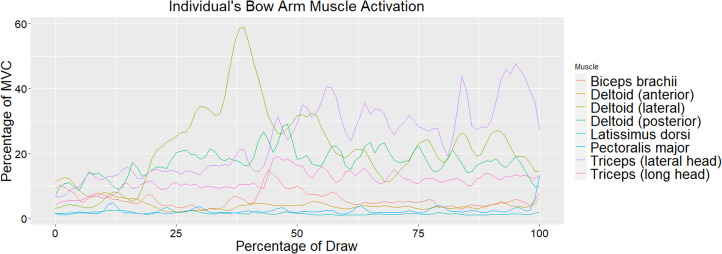
An individual's bow arm muscle activation as a percentage of MVC of the bow and draw arm as a percentage of the draw. Zero represents the start of the draw phase and 100 represents the individual at full draw.

**Figure 6.**
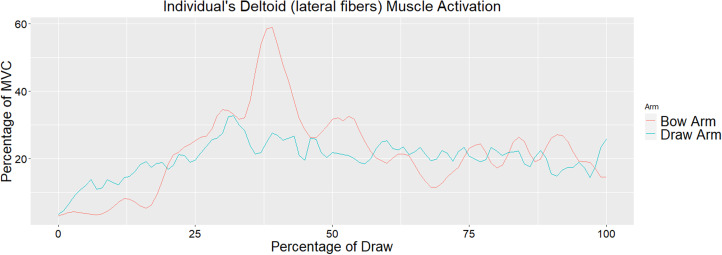
An individual's deltoid (lateral fibres) activation as a percentage of MVC of the bow and draw arm as a percentage of the draw. Zero represents the start of the draw phase and 100 represents the individual at full draw. The average for all participants shows significant difference between the bow and draw arm for iEMG and peak muscle activation.

**Figure 7.**
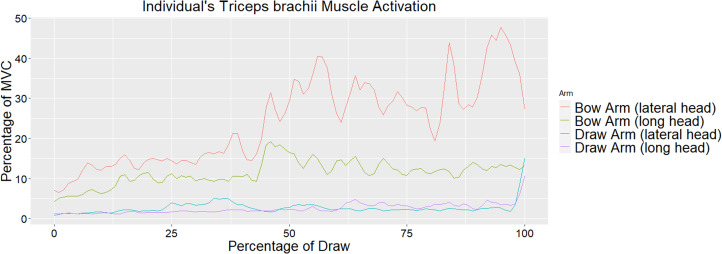
An individual's triceps brachii (lateral and long head) activation as a percentage of MVC of the bow and draw arm as a percentage of the draw. Zero represents the start of the draw phase and 100 represents the individual at full draw. The average for all participants shows significant difference between the bow and draw arm for iEMG and peak muscle activation.

Muscle activation values across all eight muscles for the draw and bow arm of one individual throughout the draw phase are seen in Figures 4 and 5, respectively. As stated earlier, the draw phase begins when the archer first begins pulling the bowstring back and ends when the archer reaches full draw. Figure 4 illustrates the high activation of the biceps brachii on the draw arm just prior to reaching 50% of draw. Figure 5 shows the high activation of the deltoid (lateral fibres) at approximately 40% of the draw and triceps (particularly the lateral head) throughout the last half of the draw. Direct comparisons between the bow and draw arm for an individual for the deltoid (lateral fibres) and triceps (lateral and long head) can be seen in Figures 6 and 7, respectively. Direct comparisons between the bow and draw arm for an individual for the remaining muscles (latissimus dorsi, posterior deltoid, anterior deltoid, pectoralis major and biceps brachii) can be found in the Supplementary Material (Figures S1–S5).

Throughout the draw phase, the movements in the draw and bow arms activate different muscles. The responsibility of the draw arm is to pull the bowstring back to create maximum tension that will propel the arrow forward upon release (Axford, 1995). Pulling the bowstring back requires elbow flexion and lateral rotation and abduction of the shoulder. Prime movers of elbow flexion include the biceps brachii and brachialis (Marieb et al., 2017). While this study did not measure activation of the brachialis, the biceps brachii activation is clearly seen on the draw arm, where it spikes in activation just prior to reaching 50% of the draw phase (Figure 4). This spike in activation represents an instant in the draw phase where the biceps brachii was recruited to help pull the bowstring back. However, it does not appear that the biceps brachii is highly activated throughout the entire draw phase for the draw arm. This is most likely due to archers using their shoulder and axial muscles to a greater extent than arm muscles to draw back the bowstring in the most efficient way possible since these muscles are larger and typically produce more power (Peterson, 1998). The lateral and posterior fibres of the deltoid are also active throughout the draw arm, but they do not appear to have as dramatic a spike in activation as the biceps brachii (Figure 4). The activation of the lateral and posterior fibres of the deltoid in the draw arm gradually increases through the first quarter of the draw phase and then remains fairly constant, with slight fluctuations around 30 and 60% of the draw phase. The more consistent activation of the deltoid muscle (particularly the posterior and lateral fibres) potentially illustrates how this archer relied more consistently on shoulder muscles to draw back the bowstring. The deltoid muscle is also a prime mover of lateral rotation and abduction of the shoulder, which are movements occurring in the draw arm throughout the draw phase (Marieb et al., 2017). It may be illuminating to observe at what point or at what time the brachialis is activated in the draw arm during the draw phase to understand whether there is a spike in activation similar to the one observed in the biceps brachii or more consistent activation throughout the draw phase.

The primary responsibility of the bow arm is to brace against the bow, requiring elbow extension and shoulder abduction (Axford, 1995). The prime mover for elbow extension is the triceps brachii muscle, while the prime mover for shoulder abduction is the deltoid muscle (Marieb et al., 2017). The most notable muscles activated in the bow arm during the draw phase are the deltoid (specifically the lateral fibres) and the triceps brachii (particularly the lateral head) (Figure 5). The lateral fibres of the deltoid spike in activation at approximately 40% of the draw phase just prior to the spike in activation observed in the biceps brachii of the draw arm. As activation of the lateral fibres of the deltoid decreases in the bow arm, around 50% of draw, activation of the triceps brachii (lateral head) increases and remains fairly high until just prior to the end of the draw phase (Figure 5). Figure 6 illustrates a direct comparison of activation of the lateral fibres of the deltoid between the draw and the bow arm. Muscle activation is similar in both arms until roughly 40% of the draw, at which point a spike in deltoid (lateral fibres) activation is seen in the bow arm, potentially a result of the increased weight of holding the bow while the shoulder is abducted. The triceps brachii show significantly more activation on the bow arm than the draw arm as a result of the required elbow extension of the bow arm. The most activation observed in the triceps is during the second half of the draw phase, suggesting that the elbow of the bow arm reaches full extension and most likely begins bracing against the bow at approximately halfway through the draw phase.

## Discussion

Increased use of bimanual technology, such as archery, is often used as an explanation for overall decreases in humeral cross-sectional asymmetry (observed in the skeletal record) resulting from increased mechanical demands placed on the non-dominant arm (Peterson, 1998; Schmitt et al., 2003). The large activation of the deltoid (lateral fibres) and triceps (lateral head) in the non-dominant bow arm is consistent with this interpretation. In addition, Rhodes and Knüsel (2005) interpreted the increased M/L humeral shape on the left, presumably non-dominant arm, of alleged archers as evidence of muscle use while holding the bow (i.e. deltoid). The large activation of the deltoid (lateral fibres) in the bow arm observed in this study supports this claim. On the other hand, the large activation of the triceps brachii muscle in elbow extension complicates this claim since extension-related movements are inferred to increase A/P bending (Schmitt et al., 2003; Rhodes & Knüsel, 2005). It is unclear to what degree deltoid and triceps brachii muscle activation would impact humeral loading. Since the deltoid has muscle fibres arranged obliquely, relative to the long axis of the muscle, there could be a decrease in the muscle force (Levangie & Norkin, 2005). This may mean that the deltoid has less influence on humeral loading. The deltoid also has an increased number of muscle fibres that could offset the decreased muscle force caused by the oblique muscle fibres (Levangie & Norkin, 2005), adding to the complexity of inferring how muscles will impact humeral morphology. The complicated relationship between muscles and bones complicates inference of specific activities based on skeletal morphology. The upper limb is especially challenging to interpret owing to the variability of movements. This research offers initial insights into how archery impacts upper arm musculature, but more research is clearly required to clarify the relationship between archery and humeral morphology.

## Limitations

For consistency, all participants in this study used the same recurve bow to avoid variation in muscle activation caused by different bow types. However, the design and construction of the bow has been modified over time (McEwen et al., 1991). The use of a modern recurve bow may involve movements and muscle activation patterns that differ from those associated with earlier bows. The original bow design could have been as simple as a tree limb bent into a D-shape arch with a bowstring, while the ends of the more complex recurve bow curve away from the archer as a way to store more energy and give the arrow more velocity (Sung et al., 2018). Changes in bow design may correspond to variation in technique and potentially variation in what muscles are activated. Data from this project reflect muscle activation while using a recurve bow and may not accurately reflect muscle activation of archers who used self-bows or longbows. Future studies should explore how muscle activation differs among bow types to address a variety of questions such as: to what extent are muscles activated based on bow typology? How does bow length impact the degree and patterns of muscle activation? How do the muscle patterns differ throughout the draw phase between older (late Pleistocene) bow designs and newer (Holocene) bow designs?

In addition to bow type, the draw weight of the bowstring could also impact upper limb musculature. This study used a constant draw weight of 0.45 kg to guarantee that each participant, regardless of experience, could fully draw the bowstring back without compromising technique or risking injury. However, bow draw weights are typically much higher. For instance, English longbows had average draw weights of approximately 125 lb (Stirland, 1992), and contemporary Olympic archers have an average bow draw weight of 48.5 lb. This large draw weight would require the archer to apply the weight of their entire body to the bow to draw back the string (Stirland, 2005). Variation in draw weight leads to the question: does increasing the draw weight change the muscles or the musculature pattern observed during the draw phase, and does increasing the draw weight increase the magnitude of the same muscles that are activated at a smaller draw weight?

Nine archers participated in this study and self-reported their experience level based on years of practising archery. Four participants reported less than one year of experience and were classified as beginners, while five participants reported more than five years of experience and were classified as experienced. Despite level of experience, all participants were recreational archers who did not report regularly practising archery throughout the year. Since experience was self-reported, there were no performance standards to qualify participants.The experienced archers in this project may be considered novice archers if performance standards were set in place. Therefore, this study should not be used to dispute research that finds significant muscle activation differences between novice and elite archers (Ertan et al., 2005; Ertan, 2009; Simsek et al., 2018).

## Future Directions

This study examined the impacts of archery on upper arm musculature but was limited to measuring only eight superficial muscles, the maximum numbers of muscles that could be measured at one time in each arm because the Delsys Trigno Wireless EMG system includes a total of 16 channels. Additional muscles, however, are presumed to be involved in archery (e.g. brachialis, brachioradialis, and coracobrachialis muscles; Peterson, 1998). The brachialis for instance, assists the biceps brachii as a prime mover in elbow flexion (Marieb et al., 2017) and could have significant activation in the draw arm. The brachialis was one of the muscles we could not measure owing to its location. Surface EMG is estimated to only be effective in recording signals ranging from 10 to 20 mm below the surface of the skin (Barkhaus & Nandedkar, 1994). Therefore, collecting muscle activation data of deeper muscles (e.g. brachialis) would require more invasive techniques, such as fine-wire electrodes. Surface EMG was used because it is non-invasive and involves minimal harmful risk to participants. When sEMG is used with small muscles (e.g. brachioradialis), discerning signals from adjacent muscles can be difficult (Kamen, 2014), limiting the quantity of available muscles for analysis. To better understand the upper arm musculature during archery, future studies should perform a more comprehensive analysis that includes all upper limb muscles.

This study examined how archery impacts musculature with EMG but did not directly measure muscle force or mechanical loading placed on the humerus. EMG can show what muscles are activated and when they are activated but does not show direct impacts on humeral morphology. Measuring muscle force itself would yield more information on the strains placed on the bone and provide a better estimate of the impact on humeral morphology. Measuring direct muscle force is invasive because it involves using force-sensing transducers that are surgically placed into the muscle (Chiu, 2018). Future analysis will apply this EMG data to a musculoskeletal model as a way of estimating muscle forces associated with archery and their direct impact on the humerus. There are mixed reviews as to whether muscle force acts as a good predictor of cross-sectional bone morphology. While muscles and bone have a close biomechanical relationship (Fricke & Schoenau, 2007), some studies suggest that bone cross-sectional geometry may not have a strong relationship with muscle force (Macintosh & Stock, 2019; Murray & Stock, 2020). For instance, Murray and Stock (2020) argue that muscular effort in the lower limb might not be enough to enhance cross-sectional properties, and that ground reaction force is also required. It would be helpful to measure both estimated muscle force (via musculoskeletal models) and humeral cross-sectional properties (via pQCT scanning) in an elite archer. This would allow for comparisons to be made between muscle activation, estimated muscle force and overall humeral morphology.

## Conclusion

The use of bows by *Homo sapiens* is speculated to have cognitive and social implications that aided in our species’ evolutionary success (Sisk & Shea, 2011; Lombard, 2011). The limitations of preservation make identifying early evidence of archery challenging. Humeral morphology has been used to support behaviour reconstructions of archery in past populations. However, the lack of experimental research concerning the impacts of archery on the upper limb weakens skeletal morphological approaches. This work aimed to contribute initial experimental research into how archery impacts upper limb musculature. While this study does not provide direct experimental validation that archery impacts humeral morphology in specific ways, it does offer insights into what muscles are activated bilaterally during archery and the temporal patterns of muscle activation during the draw phase. The relationship between archery and humeral morphology requires additional research to strengthen skeletal morphological approaches to behaviour reconstructions of past populations.

## Research transparency and reproducibility

The data that support the findings of this study are available by contacting corresponding author TD.

## Supplementary material

For supplementary material accompanying this paper visit https://doi.org/10.1017/ehs.2022.20. click here to view supplementary material
