# Supplementary material for: Archery's signature: an electromyographic analysis of the upper limb
Source: Evol Hum Sci. 2022 May 26;4:e25. doi: 10.1017/ehs.2022.20 (PMC10426064; doi:10.1017/ehs.2022.20)
Supplement: Supplementary file 1 [file S2513843X22000202sup001.docx]

|  |
| --- |

**Supplementary Material for “Archery’s signature: an electromyographic analysis of the upper limb**”

*Tabitha Dorshorst, Gillian Weir, Joseph Hamill, Brigitte Holt*

**Contents**

**Supplementary Table S1**: *Average peak muscle activation as a percentage of isometric maximum contraction (MVC).*

**Supplementary Table S2***: Average integrated EMG (iEMG) as a percentage of isometric maximum contraction (MVC).*

**Supplementary Figure S1***: An individual’s latissimus dorsi muscle activation as a percentage of MVC of the bow and draw arm as a percentage of the draw*

**Supplementary Figure S2:** *An individual’s deltoid (posterior fibers) muscle activation as a percentage of MVC of the bow and draw arm as a percentage of the draw.*

**Supplementary Figure S3:** *An individual’s deltoid (anterior fibers) muscle activation as a percentage of MVC of the bow and draw arm as a percentage of the draw*

**Supplementary Figure S4:** *An individual’s pectoralis major muscle activation as a percentage of MVC of the bow and draw arm as a percentage of the draw.*

**Supplementary Figure S5:** *An individual’s biceps brachii muscle activation as a percentage of MVC of the bow and draw arm as a percentage of the draw.*

**Summary of Results**

Supplementary Table S1 and Supplementary Table S2 show the statistically significant difference found between the dominant (draw) arm and nondominant (bow) arm for integrated EMG (iEMG) and peak amplitude muscle activation respectively. Supplementary Figures S1-S5 show a direct comparison between the draw arm and bow arm for an individual’s muscle activation through the draw phase of archery.

Average peak muscle activation values are a percentage of MVC. The draw arm refers to the arm pulling back on the string during archery and is the dominant arm, while the bow arm is the arm holding the bow and is the nondominant arm. *Statistical significance between the bow arm and the draw arm at p < 0.05.

Supplementary Table S1: Average peak muscle activation as a percentage of isometric maximum contraction (MVC)

| Peak Muscle Activation (% of MVC) | | | | | |
| --- | --- | --- | --- | --- | --- |
| Muscle | Draw Arm | | Bow Arm | |  |
|  | Mean | Std. Deviation | Mean | Std. Deviation | P-Value |
| Pectoralis Major | 9% | 0.07 | 11% | 0.10 | 0.820 |
| Latissimus Dorsi | 19% | 0.20 | 6% | 0.09 | 0.055 |
| Posterior Deltoid | 27% | 0.08 | 36% | 0.15 | 0.203 |
| Lateral Deltoid | 27% | 0.16 | 50% | 0.19 | 0.039* |
| Anterior Deltoid | 15% | 0.07 | 19% | 0.10 | 0.164 |
| Biceps Brachii | 48% | 0.23 | 20% | 0.11 | 0.019* |
| Triceps Brachii (long head) | 4% | 0.03 | 19% | 0.18 | 0.016* |
| Triceps Brachii (lateral head) | 9% | 0.05 | 46% | 0.27 | 0.016* |

Supplementary Table S2: Average integrated EMG (iEMG as a percentage of isometric maximum contraction (MVC).

| iEMG | | | | | |
| --- | --- | --- | --- | --- | --- |
| Muscle | Draw Arm | | Bow Arm | |  |
|  | Mean | Std. Deviation | Mean | Std. Deviation | P-Value |
| Pectoralis Major | 0.11 | 0.092 | 0.15 | 0.13 | 0.203 |
| Latissimus Dorsi | 0.33 | 0.33 | 0.07 | 0.06 | 0.004** |
| Posterior Deltoid | 0.47 | 0.26 | 0.71 | 0.47 | 0.164 |
| Lateral Deltoid | 0.51 | 0.33 | 1.08 | 0.62 | 0.004** |
| Anterior Deltoid | 0.23 | 0.17 | 0.39 | 0.29 | 0.301 |
| Biceps Brachii | 0.63 | 0.55 | 0.20 | 0.20 | 0.004** |
| Triceps Brachii (long head) | 0.06 | 0.04 | 0.51 | 0.65 | 0.019* |
| Triceps Brachii (lateral head) | 0.15 | 0.12 | 0.75 | 0.48 | 0.023* |

*Average iEMG values are a percentage of MVC. The draw arm refers to the arm pulling back on the string during archery and is the dominant arm, while the bow arm is the arm holding the bow and is the nondominant arm *Statistical Significance between the bow arm and the draw arm at p < 0.05, **Statistical significance between the bow arm and the draw arm at p < 0.01.*


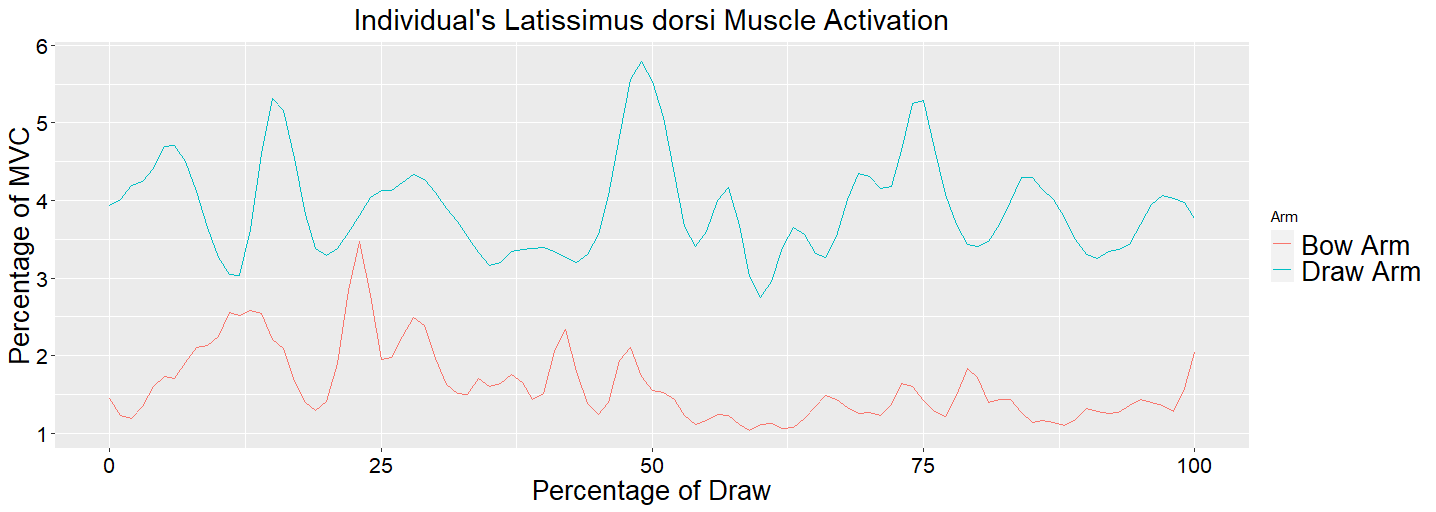


Supplementary Figure S1: An individual’s latissimus dorsi muscle activation as a percentage of MVC of the bow and draw arm as a percentage of the draw. Zero represents the start of the draw phase and 100 represents the individual at full draw. The average for all participants shows significant difference between the bow and draw arm for iEMG but not for peak muscle activation.


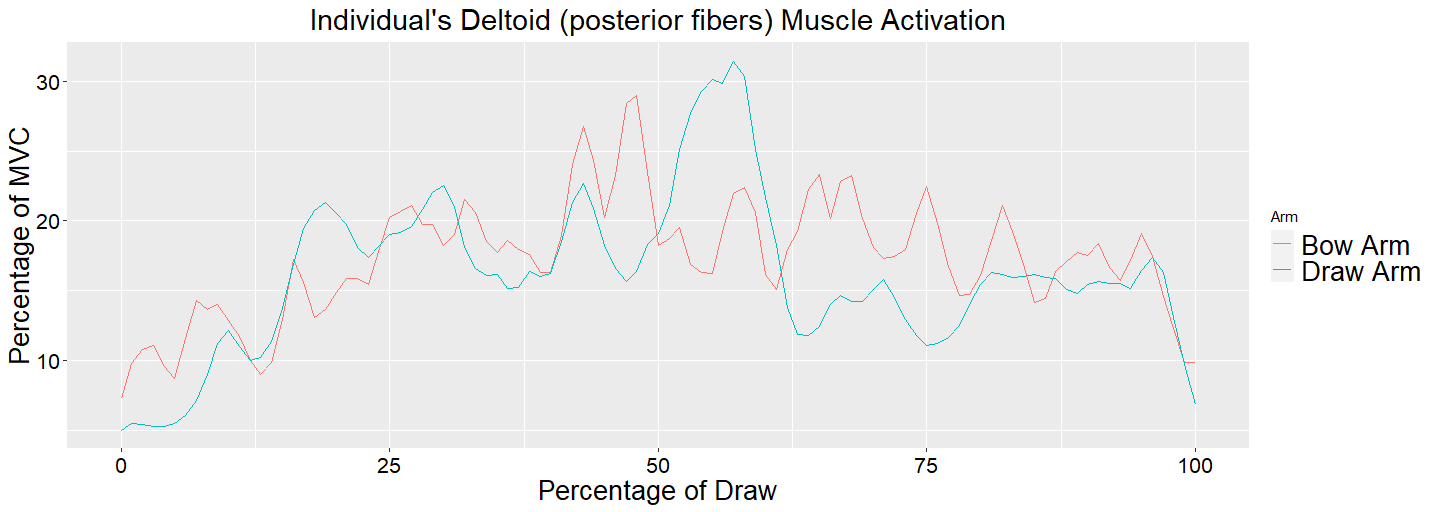


Supplementary Figure S2: An individual’s deltoid (posterior fibers) muscle activation as a percentage of MVC of the bow and draw arm as a percentage of the draw. Zero represents the start of the draw phase and 100 represents the individual at full draw. The average for all participants shows no significant difference between the bow and draw arm for iEMG or peak muscle activation.


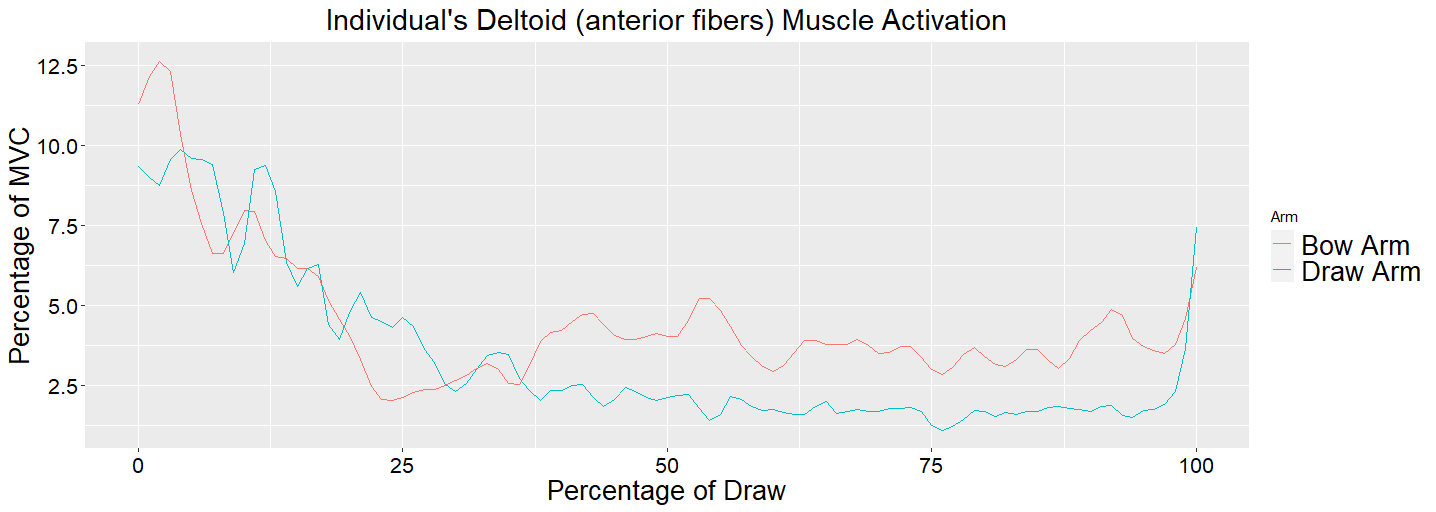


Supplementary Figure S3: An individual’s deltoid (anterior fibers) muscle activation as a percentage of MVC of the bow and draw arm as a percentage of the draw. Zero represents the start of the draw phase and 100 represents the individual at full draw. The average for all participants shows no significant difference between the bow and draw arm for iEMG or peak muscle activation.


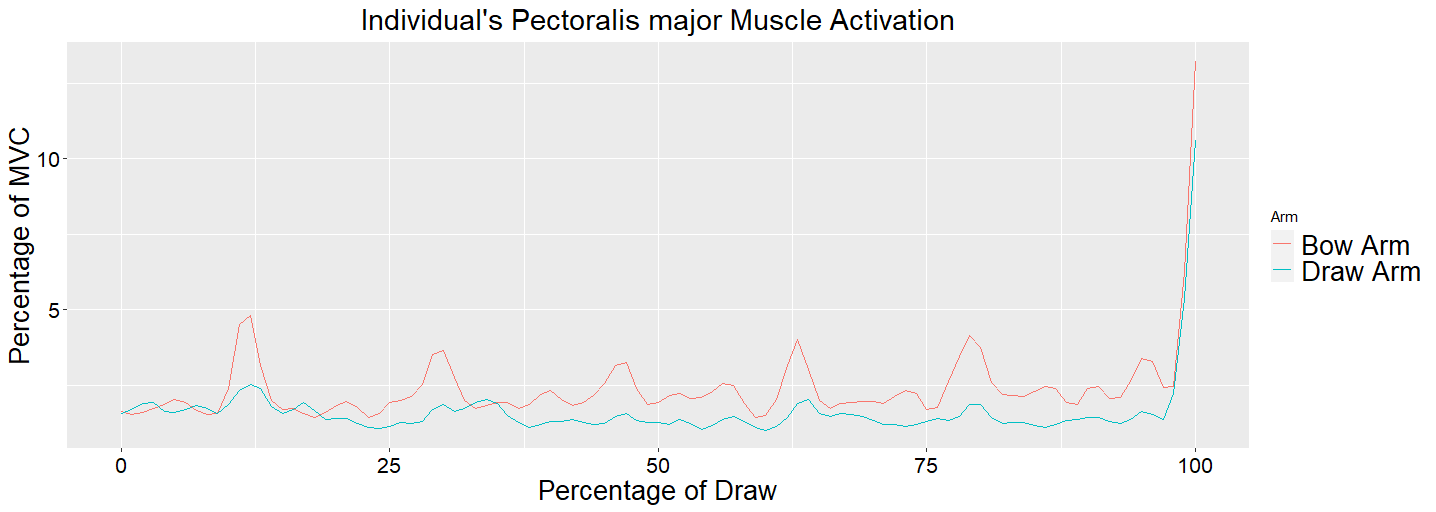


Supplementary Figure S4: An individual’s pectoralis major muscle activation as a percentage of MVC of the bow and draw arm as a percentage of the draw. Zero represents the start of the draw phase and 100 represents the individual at full draw. The average for all participants shows no significant difference between the bow and draw arm for iEMG or peak muscle activation.


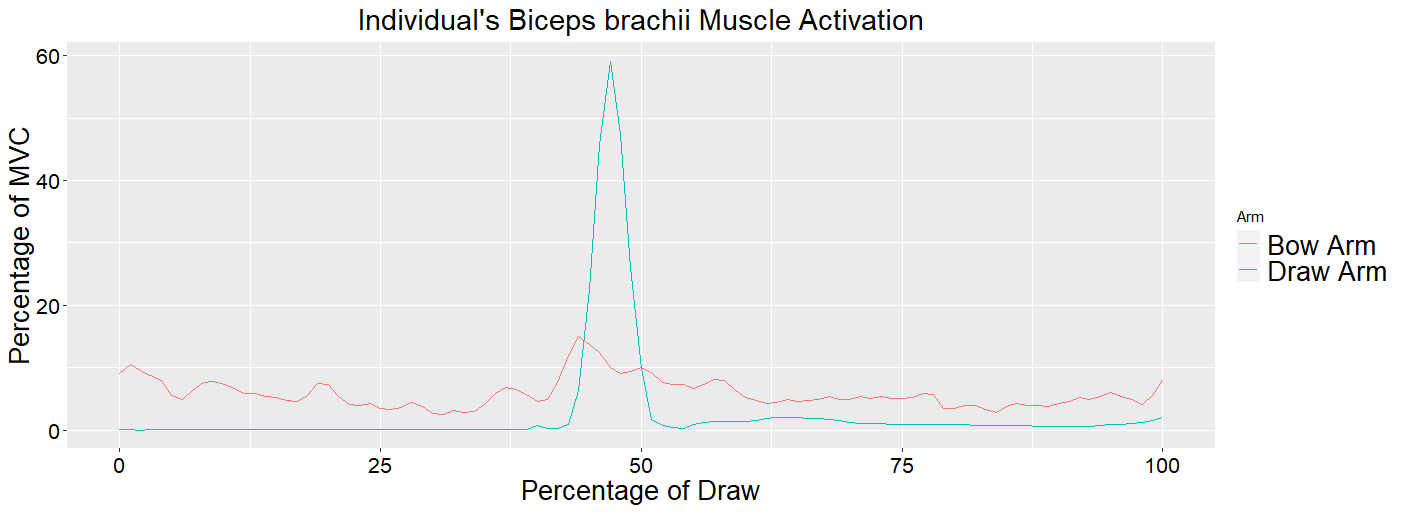


Supplementary Figure S5: An individual’s biceps brachii muscle activation as a percentage of MVC of the bow and draw arm as a percentage of the draw. Zero represents the start of the draw phase and 100 represents the individual at full draw. The average for all participants shows significant difference between the bow and draw arm for iEMG and peak muscle activation.
